# Chikungunya: a growing public health concern in the Western Pacific region

**DOI:** 10.1016/j.lanwpc.2025.101733

**Published:** 2025-10-31

**Authors:** The Lancet Regional Health – Western Pacific

China experienced its largest-ever chikungunya outbreak in Guangdong during the summer of 2025, with 16,452 locally transmitted laboratory-confirmed cases reported as of Sept 27, 2025. Before this outbreak, chikungunya had not spread widely in China, aside from a few small outbreaks linked to imported cases, with occasional sporadic local transmission in a few provinces.

Chikungunya is a mosquito-borne disease caused by the chikungunya virus and transmitted by *Aedes aegypti* and *Aedes albopictus*, the same vectors for dengue and Zika. Globally, there are an estimated 35 million chikungunya infections annually, with most cases occurring in southeast Asia, Africa, and the Americas. Although generally considered a self-limiting disease, recent studies indicate that the severity, burden, and outbreak risk of chikungunya are underestimated. Because chikungunya and dengue share transmission vectors and overlapping affected areas, there are concerns that chikungunya could follow dengue's path of global expansion, as cases have already been reported in Europe and other higher latitude areas. High fever and severe joint pain are the most common acute symptoms, and about half of patients develop chronic conditions such as joint pains that could last for years, bringing substantial social and economic impact, especially for regions like the Western Pacific with ageing populations.

In the Western Pacific region, most cases occur in tropical and subtropical territories, and non-endemic areas such as east Asia and Australia usually only see sporadic imported cases or small outbreaks. The recent outbreak in southern China likely results from a complex interplay between pathogens, vectors, environment, and hosts, demonstrating how chikungunya can rapidly escalate in dense, non-immune populations with favourable local conditions. Such events are likely to intensify in the region due to climate change expanding *Aedes* mosquito habitats, unplanned urbanisation, and increased population mobility associated with economic development.

The upscale of recent outbreaks is a warning sign that we are constantly under threat from emerging and re-emerging infectious diseases. Given the presence of driving factors for disease transmission such as different virus strains, competent vectors, and large immunologically naive populations, along with the absence of specific antiviral treatments or availability of licensed chikungunya vaccines in the Western Pacific region, there is a pressing need to assess the outbreak risks and proactively prepare for possible events in both endemic and non-endemic areas. This preparation requires coordinated surveillance and early warning systems that link vector information, health data, local knowledge, and mobility trends to identify outbreaks before they escalate. In Singapore, surveillance systems, integrated vector control strategies, and engaged local communities built for dengue control have effectively prevented severe chikungunya epidemics. As a region frequently affected by mosquito-borne diseases, a comprehensive, community-driven response that integrates existing local experience and infrastructure at various levels should be adapted and expanded to mitigate chikungunya surges.

Chikungunya is just one of many emerging and re-emerging infectious diseases present in the Western Pacific region that can exacerbate both short and long-term social, political, and economic vulnerabilities. The recent outbreak provides an opportunity to evaluate and further enhance emergency preparedness and strengthen health security systems in the region. A vital component of effective prevention and control of chikungunya and other health emergencies is the ability to swiftly adapt existing management systems to mitigate new pathogens. This requires sustained investment and regular evaluation to ensure preparedness for new threats and outbreaks, ultimately strengthening the region's capacity to withstand infectious diseases.

